# The GP Patient Survey for use in primary care in the National Health Service in the UK – development and psychometric characteristics

**DOI:** 10.1186/1471-2296-10-57

**Published:** 2009-08-22

**Authors:** John Campbell, Patten Smith, Sonja Nissen, Peter Bower, Marc Elliott, Martin Roland

**Affiliations:** 1 Peninsula Medical School, University of Exeter, Exeter, UK; 2 Ipsos MORI, London, UK; 3 NPCRDC, University of Manchester, Manchester, UK; 4 RAND Corporation, Santa Monica, USA; 5 General Practice and Primary Care Research Unit, University of Cambridge, Cambridge, UK

## Abstract

**Background:**

The UK National GP Patient Survey is one of the largest ever survey programmes of patients registered to receive primary health care, inviting five million respondents to report their experience of NHS primary healthcare. The third such annual survey (2008/9) involved the development of a new survey instrument. We describe the process of that development, and the findings of an extensive pilot survey in UK primary healthcare.

**Methods:**

The survey was developed following recognised guidelines and involved expert and stakeholder advice, cognitive testing of early versions of the survey instrument, and piloting of the questionnaire in a cross sectional pilot survey of 1,500 randomly selected individuals from the UK electoral register with two reminders to non-respondents.

**Results:**

The questionnaire comprises 66 items addressing a range of aspects of UK primary healthcare. A response rate of 590/1500 (39.3%) was obtained. Non response to individual items ranged from 0.8% to 15.3% (median 5.2%). Participants did not always follow internal branching instructions in the questionnaire although electronic controls allow for correction of this problem in analysis. There was marked skew in the distribution of responses to a number of items indicating an overall favourable impression of care. Principal components analysis of 23 items offering evaluation of various aspects of primary care identified three components (relating to doctor or nurse care, or addressing access to care) accounting for 68.3% of the variance in the sample.

**Conclusion:**

The GP Patient Survey has been carefully developed and pilot-tested. Survey findings, aggregated at practice level, will be used to inform the distribution of £65 million ($107 million) of UK NHS resource in 2008/9 and this offers the opportunity for NHS service planners and providers to take account of users' experiences of health care in planning and delivering primary healthcare in the UK.

## Background

Recent years have seen the development of patient surveys as a means of capturing patient feedback on their experience of care. The content of such surveys has generally focussed on dimensions of care reported as being of importance to patients [1,2]. In the UK NHS, the first such survey, conducted in 1998 [3], was motivated by an ambition made explicit in key strategy documents [4] that patients' views on quality of care could be taken into account to improve local services. From these early days, a national programme of NHS surveys has developed [5], capturing patients' views on a wide spectrum of their experience of healthcare. Such surveys have also formed the basis of allocating elements of NHS resource, firstly through participation in the survey process by general practitioners and primary care teams ('payment for participation'), and more recently on the basis of results obtained ('payment by results'). Following the use of questionnaires approved under the Quality and Outcomes Framework, enhanced payments for general practitioners are made to those who not only undertake practice based patient surveys, but also provide evidence of having taken action on the results of the surveys.

The national GP Patient Survey of 2007 was the first national survey of the experience of primary care patients in relation to their access to primary care. Unlike the practice based approach to surveys conducted hitherto, the 2006/7 survey used a direct (postal) approach to a sample of 5 million patients registered with all 8,472 NHS practices. Results of the survey were used to inform payments to general practitioners, with higher payments being made to practices whose patients reported greatest access to care. Following a second national GP patient survey, the UK government negotiated a revision of the survey arrangements with doctors' leaders, and proposed a revision and expansion of the survey content. We describe the development of the content of the new survey instrument for use in 2009 with revisions based on the results of the pilot survey conducted in late 2008 and reported here.

## Methods

### Mapping domains of general practice care

The broad quality framework informing the development of the survey defines quality of service provision in terms of 'access' and 'effectiveness', with the latter subdivided into 'interpersonal' and 'technical' effectiveness [6]. We mapped the aspects of general practice care which have been identified as important to patients from a number of published reviews [2,7,8]. We then reviewed a number of discrete choice experiments where patients have been asked to rank the importance of different aspects of general practice care [9-12]. We also included the requirements for the survey outlined in the Department of Health tender, which contained issues which the Department believed to be of importance to patients, as well as specific issues which were linked to payments in the general practitioner contract. As expected, there was very substantial overlap between these various sources of information on what patients value from their general practice care.

For out of hours care, we identified aspects of care that would reflect the Department of Health tender requirement of understanding, use and overall experience of out of hours services. Aspects of care in these areas were drawn largely from our previous work on out of hours care [13,14].

We specifically excluded technical aspects of care from consideration. Previous evidence suggests that patients conflate technical and interpersonal aspects of care when making judgements about technical care [15], and this is supported by more recent unpublished research in Manchester on patients' perceptions of medical errors and by empirical evidence [16] suggesting that patients' assessments may not be a sufficient basis for assessing the technical quality of their primary care. Technical aspects of care are more appropriately assessed though other mechanisms, e.g. the Quality and Outcomes Framework of the general practitioner contract.

### Identifying items for the questionnaire

We then cross referenced the attributes of general practice care valued by patients to items in a number of questionnaires commonly used in primary care in the UK, US and Europe [17-22] to identify items without copyright restrictions which might be used or adapted to meet the needs of the questionnaire. Our aim was to identify items for the new questionnaire which would reflect likely face and construct validity, and were likely to have ability to distinguish between practices with the size of sample proposed. This last criterion was different for items addressing out of hours care where data were to be reported at Primary Care Trust rather than at practice level.

The draft questionnaire was then subjected to an iterative process of development over five months which included (i) regular meetings of a joint review group, containing representatives of the academic advisors (JC and MR), staff from Ipsos MORI (including PS and SN), and representatives of the Department of Health (ii) three meetings of a stakeholder review group, including patient representatives, the British Medical Association, the Royal College of General Practitioners, the Royal College of Nursing, the Healthcare Commission, and NHS employers (iii) four waves of cognitive testing

### Cognitive testing

Four waves of cognitive testing were undertaken between July and November 2008 with progressive drafts of the questionnaire. This included a total of fifty interviews lasting between 45 and 60 minutes carried out by Ipsos MORI, with interview subjects selected to represent people from a range of socio-demographic backgrounds and people with specific types of disability (e.g. deafness) or recent experience of healthcare relevant to specific domains within the questionnaire (such as out-of-hours care).

Full details of the cognitive testing are available [23]. The interviews were conducted one-to-one and began with the respondent completing the questionnaire with an Ipsos MORI researcher present. Some respondents spontaneously mentioned issues while filling in the questionnaire while others simply completed it to the best of their ability. Once the survey had been completed the questionnaire as a whole was discussed as well as questions of interest, which were discussed in more detail.

As a result of the cognitive testing, repeated minor changes were made to the questionnaire which were then tested in the next round of cognitive interviewing. This process resulted in progressive refinement of the questionnaire over a period of five months. There were significant constraints in the development of the questionnaire in two areas. The first related to patients' ability to get an appointment within a fixed period of time (e.g. two working days). Responses to these questions were tied to ongoing payments to general practitioners as part of their contract with the NHS, and a degree of back comparability with a previous questionnaire was necessary, even though there remained some uncertainties, especially around patients' interpretation of the questions of the form 'Thinking about the last time you tried to see a doctor fairly quickly, were you able to see a doctor on the same day or in the next two days the surgery was open'.

The second area where there remained some uncertainty about patients' interpretation of the questions related to care planning. Although the UK Department of Health had made an important policy commitment to deliver written care plans to all patients with long term conditions, a significant proportion of patients found the concept difficult to interpret. The questions in this section were formulated to allow patients to express this uncertainty, with the aim that we would be able to assess over time the proportion of patients able to engage with these questions, an important issue for UK policymakers. Questions on socio-demographic aspects of care were drawn from published approved questions from the Office of National Statistics [24].

### Piloting and analysis

In November 2008, a pilot version of the questionnaire [see Additional file 1] was sent to a random sample of 1500 members of the public drawn from the electoral roll mailed second class with a covering letter. Two reminders were sent to non-responders after intervals of approximately two weeks. The results below summarise analyses of this pilot data. Except where we draw attention to differences, all items in the pilot questionnaire were identical to those in the final questionnaire [see Additional file 2].

#### (a) Response rates

In order to test the impact of questions of religion and sexuality on response rate, half of the subjects (randomly selected) received questionnaires containing these items, and half received questionnaires without them.

#### (b) Extreme and error responses

Floor and ceiling effects were investigated by inspection of the number of respondents validating extreme response categories expressed as a proportion of valid responses obtained. Errors arising from questions offering a 'branching' option were investigated by examining the number of 'error respondents' expressed as a percentage of the total number of responses in the question immediately following the question offering a branching option.

#### (c) Internal structure of the questionnaire

The internal structure of the general evaluative items (excluding items relating to care planning or out of hours care) was evaluated using exploratory principal components analysis with listwise deletion of missing variables. Inspection of a scree plot of unrotated components was used to determine the number of factors, followed by varimax rotation of the final solution to assist in the interpretation of components.

## Results

The pilot version of the questionnaire comprised 51 questions (66 items) addressing 11 domains (A-K) of healthcare (Table 1). A further domain (L) captured demographic information relating to the patient. Question format included reports and evaluations of patients' experiences; some questions comprised several items.

**Table 1:** Structure of GP patient questionnaire (pilot version)

		**Question Classification**				
**Domain**	**Section descriptor**	**Report**	**Evaluation**	**Mixed Report/Evaluation**	**Total number of questions**	**Total number of items**
A	About your GP surgery or health centre		1, 2, 4	3	4	4
B	Getting through on the phone		5		1	4
C	Seeing a doctor	6–12			7	7
D	Waiting time in the GP surgery or health centre	13	14		2	2
E	Seeing the doctor you prefer	15, 16			2	2
F	Opening hours	18,19	17		3	3
G	Seeing a doctor in the GP surgery or health centre	21	20		2	8
H	Seeing a practice nurse in the GP surgery or health centre	22	23–25		4	10
I	Care planning	26–29	30		5	5
J	Your overall satisfaction		31		1	1
K	Out of hours care	32, 33, 35	34, 36–38		7	7
L	Some questions about yourself	39–51^a^			13	13

**Total**					**51**	**66**

*Questions 5, 20, 24 have 4, 7, 7 items respectively; otherwise each question comprises only single items

^a ^Questions 50, 51 on religion and sexual identity excluded from half of pilot questionnaires

### Response rates

590/1500 patients responded (response rate 39.4%). Since the sampling frame was drawn from the electoral roll, we were not able to compare the characteristics of respondents and non-respondents in respect of age or gender. Item response profiles are available [see Additional file 3]. Missing data for each question ranged from 0.8% to 15.3% (median 5.2%, interquartile range 3.2%, 6.9%). The seventeen items in the upper quartile (>6.9%) of non response were primarily questions relating to nurse consultations, care planning for individuals with long standing health problems, the presence of long standing health problems, or sexual identity. Questionnaires incorporating questions on sexual identity and religious affiliation were associated with a similar response rate to those questionnaires not incorporating these questions (300/750, 40.0% versus 290/750, 38.7%, χ^2 ^0.28, p = 0.60).

### Extreme and error responses

**Figure 1:**
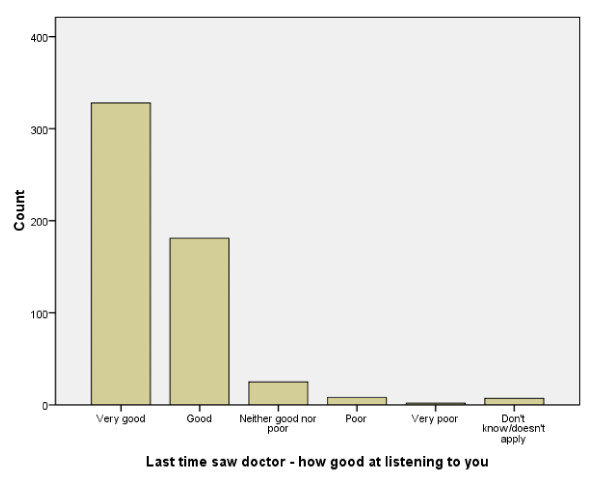
**Distribution of responses to item 'how good was the doctor at each of the following? 'Listening to you...'**.

Across the core evaluation items there was no evidence of a floor effect, but substantial evidence of skewness in responses indicative of favourable impressions of care for many items (see Figure 1 for an example relating to the item 'how good was the doctor at each of the following? 'Listening to you...').

Thirteen points in the questionnaire offered items potentially resulting in diversion ('branching') of respondents between items or sections of the questionnaire. Respondent error was evident in the 13 questions following a question offering a branch option. For example, in question 16, 398 valid responses were obtained where, on the basis of branching in question 15, only 381 respondents were eligible to respond to question 16 (and they provided 367 valid responses and 14 missing responses). The calculated error response rate was thus 31/398 (7.8%) for this question. Overall, error response rates in these 13 post-branch questions ranged from 7.8% (question 16) to 56.2% (question 28), median 16.3%. In each instance of branching error however, the error arose from respondents providing additional rather than insufficient responses where they had not followed the branching instruction. Changes were made to the final questionnaire to make the branching instructions easier to follow. However, electronic filters can readily be applied in the analysis to identify and remove such error responses in order to maintain the integrity of the denominator – the process of 'forward cleaning'.

### Internal structure of questionnaire

Principal components analysis identified three components in the 23 general evaluative items, accounting for 68.3% of the variance in the pilot sample. These components related to aspects of nurse care (eight items), doctor care (seven items), and a general component incorporating six items indicative of overall satisfaction with access arrangements (Table 2). Whilst the doctor and nurse components largely reflected contiguous items within the questionnaire, it is of note that an item addressing the ease of getting an appointment with the practice nurse (question 23) was contiguous with other items reflecting doctor or nurse care, but actually loaded with other non-contiguous items in the access component. This suggests that the factor structure is not simply a reflection of the order of the questions in the survey. The global item addressing overall satisfaction with care (question 31) additionally loaded on all three components, but had a higher loading on the access component, rather than onto the items reflecting inter-personal aspects of doctor or nurse care. The item addressing the physical accessibility of practice premises did not load onto any of the three identified components.

**Table 2:** Loadings of principal components of national GP patient survey evaluative items (n = 23) after excluding those sections items not relating to general primary care (bolded items load > 0.3)

	**Component**		
	1	2	3

q1 Getting into the building	0.110	-0.023	0.230
q2 Cleanliness of GP surgery	0.065	0.154	**0.332**
q4 Helpfulness of receptionists	0.054	0.150	**0.597**
q5 Getting through on the phone	0.025	0.072	**0.623**
q14 How feel about how long wait	0.194	0.128	**0.496**
q17 Satisfaction with opening hours	0.073	0.218	**0.575**
q20 Last time saw doctor – how good at giving you enough time	0.016	**0.768**	0.175
q20 Last time saw doctor – how good at asking about your symptoms	0.160	**0.852**	0.143
q20 Last time saw doctor – how good at listening to you	0.191	**0.877**	0.135
q20 Last time saw doctor – how good at explaining tests and treatments	0.243	**0.780**	0.211
q20 Last time saw doctor – how good at involving you in decisions	0.212	**0.809**	0.214
q20 Last time saw doctor – how good at treating you with care and concern	0.200	**0.886**	0.192
q20 Last time saw doctor – how good at taking your problems seriously	0.184	**0.838**	0.214
q23 Getting an appointment with practice nurse	0.235	0.141	**0.409**
q24 Last time saw practice nurse – how good at giving you enough time	**0.694**	0.210	0.222
q24 Last time saw practice nurse – how good at asking about your symptoms	**0.886**	0.279	0.190
q24 Last time saw practice nurse – how good at listening to you	**0.926**	0.176	0.152
q24 Last time saw practice nurse – how good at explaining tests and treatments	**0.913**	0.183	0.195
q24 Last time saw practice nurse – how good at involving you in decisions	**0.918**	0.188	0.211
q24 Last time saw practice nurse – how good at treating you with care and concern	**0.940**	0.110	0.102
q24 Last time saw practice nurse – how good at taking your problems seriously	**0.952**	0.132	0.152
q25 Overall quality of care provided by practice nurse	**0.836**	0.117	0.224
q31 Satisfaction with care at GP surgery	**0.342**	**0.400**	**0.630**

A number of modifications were made to the wording and presentation of questions, and to the overall presentation of the questions and items. The final agreed wording and presentation of the questionnaire [see Additional file 2] was distributed to patients in January 2009.

## Discussion

The national General Practice Patient Survey is one of the largest annual surveys of patients' experience of health care to take place anywhere in the world. The survey provides a snapshot overview of the quality of care provided by nearly 9,000 UK general practices. The results of the survey will inform the allocation of around £65 million ($107 million) of general practitioners' remuneration, and provide a basis for informing service delivery of UK primary healthcare.

The survey has been carefully developed, following recognised guidelines [25] relevant to healthcare survey development involving expert and stakeholder advice, cognitive testing of early versions of the survey instrument, and piloting of the questionnaire in a substantial number of adults registered to receive NHS care. In developing this instrument, we drew on the content of a range of questionnaires designed as postal surveys from the UK and elsewhere. We elected not to include in our review a number of other important survey instruments where these were principally designed for post-consultation distribution. We recognise that this led to the omission of certain domains of relevance to primary care, such as enablement [26] and empathy [27]. There is also a tension between the desire to meet the needs of multiple stakeholders, and the need to ensure that the survey is useable and that response rates are not reduced by survey length.

Items include both patient self reports of their recent experiences of primary health care, as well as evaluations of that care. Although questioned by some [28], we believe that both of these types of questions add value to the information obtained as a result of conducting the survey and, as report-evaluation pairs, may offer specific value in contributing to standard setting in respect of primary care [29].

Testing and evaluation of surveys is a complex and ongoing process rather than being based on a single study. Following the pilot, extensive validity and reliability testing will be undertaken and reported, drawing on data specifically obtained to investigate internal consistency and test-retest reliability, as well as various facets of validity, especially construct and predictive validity. For example, concerns have been raised about the ability of patients to recall their experience of access up to 6 months previously. Although evidence from economic analyses suggest that patients are reasonably accurate reporters of aspects of health care utilisation [30,31], the validity of these items in the context of this survey remains to be tested.

Although a modest response rate was evident after two reminders, this is in line with previous large scale surveys in which unsolicited responses are invited [32]. Non-response is an issue if non-respondents differ from respondents on the key measures of interest. Although we cannot estimate the magnitude of any such bias for the pilot sample, recent meta-analyses suggest that as long as rigorous probability sample processes are followed (such as those proposed for the main survey), the association between response rates and non-response bias within samples is generally weak [33,34].

Non-response is likely to be an issue when considering the use of surveys in quality improvement activities and in relation to financial reimbursement, and more sophisticated analyses are planned when the first full surveys are completed. The present research however, focussed on the basic performance of the instrument per se, where non-response bias is less of an issue.

However, non-response bias can still influence the survey performance measures described in the present paper. For example, the evidence of skew reported here may be accentuated by non-response (if patients with poor experiences are less likely to respond), and the levels of missing data reported may be low because respondents are more likely to be literate and used to completing forms. It is also possible that aspects of the design of the instrument (such as overall length) have an impact on response rates. The skewness observed in responses to a number of items are common in patient reports and evaluations of health care [35] and do not necessarily limit the ability to reliably distinguish practices and patient subgroups with sufficient sample sizes [36].

The questionnaire adopts an internal branching structure whereby respondents are directed through the items in a way which is cognisant of their experience of care. Thus, not all respondents were expected to answer every question. However, branching error was evident in the pilot presentation of some items, and on this account, clearer signposting between branch items was introduced for the final version of the questionnaire.

Principal components analysis of the survey instrument reported here identified three components contributing to the internal structure of the general evaluative items in the questionnaire. These components relate to care provided either by a doctor or a nurse, as well as a component comprising items focussing on the accessibility of primary care services. The high loadings associated with the interpersonal scale items in the nursing scale might reflect redundancy, and there might be potential for reducing the length of the survey. However, this might impact on content validity (if the full range of interpersonal care issues are not adequately sampled) and utility, as professionals may wish to have a range of items to more accurately measure their individual skills and provide a more fine-grained assessment for training purposes.

In this pilot survey, the higher loading of an item relating to overall satisfaction with care onto the access component provides some support to UK government policy which incentivises prompt access to care over either interpersonal care or factors relating to personal continuity of care. However, it should be noted that the satisfaction item had a reasonably high loading across all three dimensions, and the results of discrete choice experiments do suggest that any global focus on access needs to take into account the fact that the value of access is contingent on a number of factors, including patient characteristics and the type of problem being presented [9-12]. It is important that policy makers are attuned to these variable results and do not accord undue priority to global survey results.

The inclusion of two questions addressing the areas of religious belief and sexual identity was not associated with any adverse effect on overall survey response rates. Where these questions were included however, it may be of note that five times as many individuals failed to respond to the question on sexual identity compared to the question on religious belief, suggesting that the latter may be more acceptable to potential respondents than the former. These questions were not included in the 2008/09 version of the final questionnaire, but will be included in subsequent years to allow assessment of inequality issues.

## Conclusion

The final version of the survey is provided in 15 languages, in Braille, in British Sign Language, in both paper and on-line formats, and is supported by online and telephone resources. Following modifications, the final questionnaire was mailed to 5.6 million randomly selected patients from UK GP lists in January 2009. The results of that survey aggregated at practice level will be published in mid 2009. Research relating to the main survey data is planned.

## Competing interests

The GP Patient Survey was commissioned by the UK Department of Health following an open tendering process. The survey is administered by Ipsos MORI UK Ltd. JC and MR are scientific advisors to the survey.

## Authors' contributions

All authors except ME were involved in the development of the GP Patient Survey. SN managed the cognitive testing component, and administered the pilot survey. JC conducted the analysis and wrote the paper. All authors read and approved the final manuscript.

## Pre-publication history

The pre-publication history for this paper can be accessed here:



## Supplementary Material

Additional file 1 **Pilot GPPS version**. Pilot version of the GPPS scale. Click here for file

Additional file 2 **Final GPPS version**. Final version of the GPPS scale. Click here for file

Additional file 3 **Survey responses**. Responses to GP patient survey questions. Click here for file
