# Supplementary material for: The GP Patient Survey for use in primary care in the National Health Service in the UK – development and psychometric characteristics
Source: BMC Fam Pract. 2009 Aug 22;10:57. doi: 10.1186/1471-2296-10-57 (PMC2736918; doi:10.1186/1471-2296-10-57)
Supplement: Additional file 2 — Final GPPS version. Final version of the GPPS scale. [file 1471-2296-10-57-S2.doc]

**National GP patient survey (final version for use 2008/9)**
