# Supplementary material for: The GP Patient Survey for use in primary care in the National Health Service in the UK – development and psychometric characteristics
Source: BMC Fam Pract. 2009 Aug 22;10:57. doi: 10.1186/1471-2296-10-57 (PMC2736918; doi:10.1186/1471-2296-10-57)
Supplement: Additional file 3 — Survey responses. Responses to GP patient survey questions. [file 1471-2296-10-57-S3.doc]

Responses to GP patient survey questions (590 respondents). Extended response profiles (including floor and ceiling effects, numbers of ‘haven’t tried and ’don’t know’ responses) are provided for questions with evaluative component

| **Qn number** | **Item number** | **Descriptor; >Potential diversion to branch question (n selecting branch option)** | **Eligible respondents (n)[[1]](#footnote-2)** | **Valid Responses obtained (n)** | **Multicode responses (n)** | **[[2]](#footnote-3)Missing (n)** | **Missing as % eligible respondents** | **Floor effects (n)[[3]](#footnote-4)** | **Valid responses: % floor** |  | **Ceiling effects (n)[[4]](#footnote-5)** | **Valid responses: % ceiling** |  | **'Haven't tried' (n)** | **‘Haven’t tried’ as % valid responses** |  | **‘don’t know’ (n)** | **‘Don’t knmow’ as % valid responses** |
| --- | --- | --- | --- | --- | --- | --- | --- | --- | --- | --- | --- | --- | --- | --- | --- | --- | --- | --- |
|  |  |  |  |  |  |  |  |  |  |  |  |  |  |  |  |  |  |  |
| 1 | 1 | Getting into the building | 590 | 581 | 1 | 8 | 1.4 | 4 | 0.7 |  | 476 | 81.9 |  |  |  |  |  |  |
| 2 | 2 | Cleanliness of GP surgery | 590 | 582 | 3 | 5 | 0.8 | 0 | 0.0 |  | 451 | 77.5 |  |  |  |  | 9 | 1.5 |
| 3 | 3 | Can other patients overhear in reception | 590 | 572 | 4 | 14 | 2.4 |  |  |  |  |  |  |  |  |  | 44 | 7.7 |
| 4 | 4 | Helpfulness of receptionists | 590 | 575 | 1 | 14 | 2.4 | 1 | 1.2 |  | 61 | 72.6 |  |  |  |  |  |  |
| 5 | 5 | Getting through on the phone | 590 | 571 | 3 | 16 | 2.7 | 38 | 6.7 |  | 170 | 29.8 |  | 50 | 8.8 |  | 11 | 1.9 |
| 5 | 6 | Speaking to a doctor on the phone | 590 | 557 | 1 | 32 | 5.4 | 32 | 5.7 |  | 60 | 10.8 |  | 246 | 44.2 |  | 69 | 12.4 |
| 5 | 7 | Speaking to a nurse on the phone | 590 | 561 | 1 | 28 | 4.7 | 18 | 3.2 |  | 75 | 13.4 |  | 258 | 46.0 |  | 89 | 15.9 |
| 5 | 8 | Getting test results on the phone | 590 | 560 | 2 | 28 | 4.7 | 26 | 4.6 |  | 107 | 19.1 |  | 231 | 41.3 |  | 73 | 13.0 |
| 6 | 9 | Tried to see a doctor within 48 hours >9 (194) | 590 | 551 | 0 | 39 | 6.6 |  |  |  |  |  |  |  |  |  |  |  |
| 7 | 10 | When actually seen >9 (274) | 357 | 333 | 6 | 18 | 5.0 |  |  |  |  |  |  |  |  |  |  |  |
| 8 | 11 | Why couldn't be seen within 48 hours | 59 | 58 | - | 1 | 1.7 |  |  |  |  |  |  |  |  |  |  |  |
| 9 | 12 | Tried to book ahead >11 (283) | 590 | 552 | 0 | 38 | 6.4 |  |  |  |  |  |  |  |  |  |  |  |
| 10 | 13 | Offered a book ahead appointment | 269 | 254 | 1 | 14 | 5.2 |  |  |  |  |  |  |  |  |  |  |  |
| 11 | 14 | When last saw a doctor at GP surgery >13 (448) | 590 | 558 | 1 | 31 | 5.3 |  |  |  |  |  |  |  |  |  |  |  |
| 12 | 15 | Why haven't seen a doctor in past 6 months | 110 | 100 | 1 | 9 | 8.2 |  |  |  |  |  |  |  |  |  |  |  |
| 13 | 16 | How long have to wait to be seen | 590 | 552 | 11 | 27 | 4.6 |  |  |  |  |  |  |  |  |  |  |  |
| 14 | 17 | How feel about how long wait | 590 | 530 | 3 | 57 | 9.7 | 27 | 5.1 |  | 343 | 64.7 |  |  |  |  | 39 | 7.4 |
| 15 | 18 | Particular doctor prefer to see >17 (176) | 590 | 557 | 1 | 32 | 5.4 |  |  |  |  |  |  |  |  |  |  |  |
| 16 | 19 | How often see preferred doctor | 381 | 367 | 0 | 14 | 3.7 |  |  |  |  |  |  |  |  |  |  |  |
| 17 | 20 | Satisfaction with opening hours | 590 | 565 | 0 | 25 | 4.2 | 6 | 1.1 |  | 241 | 42.7 |  |  |  |  | 15 | 2.7 |
| 18 | 21 | Want additional opening hours >20 (287) | 590 | 528 | 0 | 62 | 10.5 |  |  |  |  |  |  |  |  |  |  |  |
| 19 | 22 | What additional opening hours would you most like surgery to be open | 241 | 200 | 35 | 6 | 2.5 |  |  |  |  |  |  |  |  |  |  |  |
| 20 | 23 | Last time saw doctor - how good at giving you enough time | 590 | 570 | 1 | 19 | 3.2 | 3 | 0.5 |  | 348 | 61.1 |  |  |  |  | 8 | 1.4 |
| 20 | 24 | Last time saw doctor - how good at asking about your symptoms | 590 | 552 | 0 | 38 | 6.4 | 3 | 0.5 |  | 314 | 56.9 |  |  |  |  | 15 | 2.7 |
| 20 | 25 | Last time saw doctor - how good at listening to you | 590 | 551 | 0 | 39 | 6.6 | 2 | 0.4 |  | 328 | 59.5 |  |  |  |  | 7 | 1.3 |
| 20 | 26 | Last time saw doctor - how good at explaining tests and treatments | 590 | 554 | 1 | 35 | 5.9 | 1 | 0.2 |  | 292 | 52.7 |  |  |  |  | 48 | 8.7 |
| 20 | 27 | Last time saw doctor - how good at involving you in decisions | 590 | 550 | 0 | 40 | 6.8 | 5 | 0.9 |  | 266 | 48.4 |  |  |  |  | 56 | 10.2 |
| 20 | 28 | Last time saw doctor - how good at treating you with care and concern | 590 | 555 | 2 | 33 | 5.6 | 4 | 0.7 |  | 314 | 56.6 |  |  |  |  | 18 | 3.2 |
| 20 | 29 | Last time saw doctor - how good at taking your problems seriously | 590 | 556 | 1 | 33 | 5.6 | 6 | 1.1 |  | 316 | 56.8 |  |  |  |  | 22 | 4.0 |
| 21 | 30 | Confidence and trust in doctor | 590 | 569 | 1 | 20 | 3.4 |  |  |  |  |  |  |  |  |  | 7 | 1.2 |
| 22 | 31 | Seen a practice nurse in past 6 months, >26 (241) | 590 | 573 | 0 | 17 | 2.9 |  |  |  |  |  |  |  |  |  |  | 0.0 |
| 23 | 32 | Getting an appointment with practice nurse | 332 | 305 | 0 | 27 | 8.1 | 2 | 0.7 |  | 179 | 58.7 |  | 18 | 5.9 |  | 6 | 2.0 |
| 24 | 33 | Last time saw practice nurse - how good at giving you enough time | 332 | 310 | 1 | 21 | 6.3 | 1 | 0.3 |  | 214 | 69.0 |  |  |  |  | 11 | 3.5 |
| 24 | 34 | Last time saw practice nurse - how good at asking about your symptoms | 332 | 300 | 0 | 32 | 9.6 | 2 | 0.7 |  | 172 | 57.3 |  |  |  |  | 30 | 10.0 |
| 24 | 35 | Last time saw practice nurse - how good at listening to you | 332 | 301 | 0 | 31 | 9.3 | 2 | 0.7 |  | 189 | 62.8 |  |  |  |  | 10 | 3.3 |
| 24 | 36 | Last time saw practice nurse - how good at explaining tests and treatments | 332 | 297 | 1 | 34 | 10.2 | 4 | 1.3 |  | 173 | 58.2 |  |  |  |  | 24 | 8.1 |
| 24 | 37 | Last time saw practice nurse - how good at involving you in decisions | 332 | 294 | 0 | 38 | 11.4 | 4 | 1.4 |  | 158 | 53.7 |  |  |  |  | 40 | 13.6 |
| 24 | 38 | Last time saw practice nurse - how good at treating you with care and concern | 332 | 300 | 1 | 31 | 9.3 | 4 | 1.3 |  | 190 | 63.3 |  |  |  |  | 13 | 4.3 |
| 24 | 39 | Last time saw practice nurse - how good at taking your problems seriously | 332 | 300 | 1 | 31 | 9.3 | 4 | 1.3 |  | 179 | 59.7 |  |  |  |  | 26 | 8.7 |
| 25 | 40 | Overall quality of care provided by practice nurse | 332 | 312 | 0 | 20 | 6.0 | 3 | 1.0 |  | 225 | 72.1 |  |  |  |  | 4 | 1.3 |
| 26 | 41 | Long-standing health problem, disability or infirmity >31(260) | 590 | 563 | 0 | 27 | 4.6 |  |  |  |  |  |  |  |  |  | 13 | 2.3 |
| 27 | 42 | Had a discussion about managing health and care needs >29 (201) | 303 | 288 | 0 | 15 | 5.0 |  |  |  |  |  |  |  |  |  | 7 | 2.4 |
| 28 | 43 | Offered chance to discuss managing health and care needs >31 (87) | 87 | 74 | 0 | 13 | 14.9 |  |  |  |  |  |  |  |  |  | 22 | 29.7 |
| 29 | 44 | Did you agree a plan about managing health and care needs | 201 | 186 | 0 | 15 | 7.5 |  |  |  |  |  |  |  |  |  | 17 | 9.1 |
| 30 | 45 | Did having a discussion help improve the care you receive | 201 | 181 | 0 | 20 | 10.0 |  |  |  |  |  |  |  |  |  | 23 | 12.7 |
| 31 | 46 | Satisfaction with care at GP surgery | 590 | 575 | 0 | 15 | 2.5 | 4 | 0.7 |  | 350 | 59.3 |  |  |  |  |  |  |
| 32 | 47 | Know how to contact an out-of-hours GP when surgery closed | 590 | 570 | 0 | 20 | 3.4 |  |  |  |  |  |  |  |  |  |  |  |
| 33 | 48 | Tried to contact an out-of-hours GP when surgery was closed in past 6 months >39 (473) | 590 | 559 | 3 | 28 | 4.7 |  |  |  |  |  |  |  |  |  |  |  |
| 34 | 49 | How easy to make contact with out-of-hours service | 86 | 84 | 0 | 2 | 2.3 | 3 | 3.5 |  | 41 | 48.8 |  |  |  |  |  |  |
| 35 | 50 | Prescribed or recommended medicines by out-of-hours GP >37 (36) | 86 | 82 | 0 | 4 | 4.7 |  |  |  |  |  |  |  |  |  |  |  |
| 36 | 51 | How easy was it to get medicines | 46 | 44 | 0 | 2 | 4.3 | 3 | 6,8 |  | 14 | 31.8 |  |  |  |  |  |  |
| 37 | 52 | How do you feel about how quickly you received care out-of-hours | 86 | 80 | 0 | 6 | 7.0 | 20 | 25.0 |  | 60 | 75.0 |  |  |  |  |  |  |
| 38 | 53 | Overall, how do you feel about the care you received out-of-hours | 86 | 81 | 0 | 5 | 5.8 | 1 | 1.2 |  | 32 | 39.5 |  |  |  |  |  |  |
| 39 | 54 | Gender | 590 | 572 | 0 | 18 | 3.1 |  |  |  |  |  |  |  |  |  |  |  |
| 40 | 55 | Age | 590 | 571 | 0 | 19 | 3.2 |  |  |  |  |  |  |  |  |  |  |  |
| 41 | 56 | Ethnic group | 590 | 547 | 24 | 19 | 3.2 |  |  |  |  |  |  |  |  |  |  |  |
| 42 | 57 | Which of these describes what you are doing at present >45 (282) | 590 | 537 | 14 | 39 | 6.6 |  |  |  |  |  |  |  |  |  |  |  |
| 43 | 58 | Journey time from home to work | 255 | 250 | 1 | 4 | 1.6 |  |  |  |  |  |  |  |  |  |  |  |
| 44 | 59 | Could you see a GP during working hours if you needed to | 255 | 246 | 1 | 8 | 3.1 |  |  |  |  |  |  |  |  |  |  |  |
| 45 | 60 | Long-standing health problems or disabilities | 590 | 429 | 71 | 90 | 15.3 |  |  |  |  |  |  |  |  |  |  |  |
| 46 | 61 | Deaf person who uses sign language | 590 | 514 | 2 | 74 | 12.5 |  |  |  |  |  |  |  |  |  |  |  |
| 47 | 62 | General health status | 590 | 575 | 3 | 12 | 2.0 |  |  |  |  |  |  |  |  |  |  |  |
| 48 | 63 | Parent or legal guardian of child under 16 | 590 | 552 | 0 | 38 | 6.4 |  |  |  |  |  |  |  |  |  |  |  |
| 49 | 64 | Carer responsibilities for anyone in household | 590 | 566 | 0 | 24 | 4.1 |  |  |  |  |  |  |  |  |  |  |  |
| 50 | 65 | Sexual orientation | 290 | 264 | 1 | 25 | 8.6 |  |  |  |  |  |  |  |  |  |  |  |
| 51 | 66 | Religion | 290 | 283 | 2 | 5 | 1.7 |  |  |  |  |  |  |  |  |  |  |  |

1. Eligibility determined by response to preceding questions , some of which contained branching options (see text and Figures) [↑](#footnote-ref-2)
2. ^M^ [↑](#footnote-ref-3)
3. Respondents validating the lowest response option (reflecting adverse rating) [↑](#footnote-ref-4)
4. Respondents validating the highest response option (reflecting favourable rating) [↑](#footnote-ref-5)
